# Aromatase Inhibitor-Associated Musculoskeletal Syndrome: Understanding Mechanisms and Management

**DOI:** 10.3389/fendo.2021.713700

**Published:** 2021-07-27

**Authors:** Tara Hyder, Christopher C. Marino, Sasha Ahmad, Azadeh Nasrazadani, Adam M. Brufsky

**Affiliations:** ^1^University of Pittsburgh Physicians, University of Pittsburgh Medical Center, Pittsburgh, PA, United States; ^2^Mario Lemieux Center for Blood Cancers, University of Pittsburgh Medical Center (UPMC) Hillman Cancer Center, Pittsburgh, PA, United States; ^3^Department of Sciences, Sewickley Academy, Pittsburgh, PA, United States; ^4^UPMC Hillman Cancer Center, Magee Women’s Hospital, Pittsburgh, PA, United States

**Keywords:** aromatase inhibitor, aromatase inhibitor-associated musculoskeletal syndrome, breast cancer, aromatase inhibitor-induced arthralgia, aromatase inhibitor-induced bone loss

## Abstract

Aromatase inhibitors (AIs) are a key component in the chemoprevention and treatment of hormone receptor-positive (HR+) breast cancer. While the addition of AI therapy has improved cancer-related outcomes in the management of HR+ breast cancer, AIs are associated with musculoskeletal adverse effects known as the aromatase inhibitor-associated musculoskeletal syndrome (AIMSS) that limit its tolerability and use. AIMSS is mainly comprised of AI-associated bone loss and arthralgias that affect up to half of women on AI therapy and detrimentally impact patient quality of life and treatment adherence. The pathophysiology of AIMSS is not fully understood though has been proposed to be related to estrogen deprivation within the musculoskeletal and nervous systems. This review aims to characterize the prevalence, risk factors, and clinical features of AIMSS, and explore the syndrome’s underlying mechanisms and management strategies.

## Introduction

HR+ breast cancer is the most common subtype of breast cancer and a significant cause of cancer-related death in women (1). Endocrine therapy targeting aromatase has an important role in the primary chemoprevention of HR+ breast cancer in high-risk postmenopausal women and is integral to the management of HR+ breast cancer in the adjuvant and metastatic setting to prevent recurrence and control progression of disease, respectively.

Aromatase is a cytochrome p450 enzyme encoded by the CYP19A1 gene that converts androgens to estrogens, specifically converting testosterone and androstenedione to aromatic estrogens estradiol and estrone, respectively (2). Aromatase facilitated production of estrogens primarily occurs in the ovaries of premenopausal women, whereas in postmenopausal women it takes place in peripheral tissues, particularly adipose tissue. Aromatase has been found to be expressed in numerous tissues including placenta, the central nervous system, bone, muscle, testis, prostate, adrenals, and skin facilitating pleiotropic roles in the body (3). In HR+ breast cancer, aromatase is frequently overexpressed in breast endothelial cells and the surrounding stroma leading to local estrogen synthesis within the tumor microenvironment, thus stimulating cancer growth through estrogen receptor activation (4). Lifetime exposure to estrogen correlates with breast cancer risk by promoting carcinogenesis and the proliferation of neoplastic breast tissue (5, 6). The essential role of aromatase in estrogen production makes it an ideal target for endocrine therapy in the management of HR+ breast cancer.

The advent of third generation aromatase inhibitors (AIs) has played a significant role in advancing the endocrine chemoprevention and treatment of HR+ breast cancer. At present, there are three AIs in common practice. Anastrozole and letrozole are nonsteroidal AIs that competitively inhibit aromatase, whereas exemestane is a steroidal AI that irreversibly binds and inhibits aromatase (3, 7, 8). Clinical trials have demonstrated a role for AIs in breast cancer chemoprevention and have been shown to reduce the rate of breast cancer development in high-risk women. In the MAP.3 trial, high-risk postmenopausal women received either exemestane or placebo (9). After a median follow up of 35 months, there was a 53% reduction in the incidence of all breast cancer and a 65% reduction in invasive breast cancer. The International Breast Cancer Intervention Study II (IBIS-II) randomized high-risk postmenopausal women with no breast cancer to receive either anastrozole or placebo and found a significant reduction in incidence for all breast cancer (53%), including invasive HR+ breast cancer (58%) and ductal carcinoma *in situ* (70%) in the anastrozole group (10). A second analysis at 131 months identified continuing reduction in breast cancer with anastrozole (11). AIs have also demonstrated clinical superiority over tamoxifen in postmenopausal women and have established themselves as standard of care in both the adjuvant and metastatic setting (12–14). Multiple trials have demonstrated improved outcomes in postmenopausal women who switched from tamoxifen to an AI for extended endocrine therapy in the adjuvant setting (15–19). Adjuvant AI therapy is presently recommended for five years to prevent recurrence though AI therapy extended to 10 years can provide further disease-free survival in certain high-risk individuals (20, 21). Moreover, growing evidence has supported the role of AIs in premenopausal women at high risk for disease recurrence when combined with ovarian function suppression (22–24). Over two decades of clinical experience, AI therapy has proven itself to be a major achievement in breast cancer endocrine therapy.

While generally considered to have a well-tolerated side effect profile (25), AIs have been recognized to cause musculoskeletal symptoms resulting in diminished quality of life and frequent discontinuation of therapy (26). Musculoskeletal symptoms of AIs that have been described include arthralgias, myalgias, joint stiffness, and tendinopathy (27, 28). Furthermore, AIs appear to augment bone mineral density decline observed during menopause (29). The constellation of these musculoskeletal symptoms has become known as the AI-associated musculoskeletal syndrome (AIMSS). Given the established benefits of AI treatment, this review aims to summarize the current knowledge about the prevalence, pathophysiology, and management strategies for AIMSS.

## Prevalence and Associated Risk Factors

The reported prevalence of AIMSS varies widely in the literature but is estimated to occur in one-third to one-half of women utilizing AIs (30–32). One meta-analysis involving 21 studies with 13,177 participants found a prevalence of arthralgia in women on AI therapy ranging from 20-74% with a pooled estimate of 46% (30). Even in the preventative setting where AIs have demonstrated a significant reduction in breast cancer incidence, there was an increased frequency of grade 2 or higher symptoms of AIMSS including arthralgias, myalgias, joint stiffness, and carpal tunnel syndrome (9, 33). Whereas the reported AIMSS prevalence varies considerably, the true prevalence has been suggested to be higher than initially appreciated in early clinical trials (31, 34).

AIMSS frequently leads to early discontinuation and nonadherence of AI therapy in a significant proportion of patients, which in turn has been associated with breast cancer recurrence and increased all-cause mortality (35). One study demonstrated that breast cancer patients who discontinued endocrine therapy had a 20% recurrence of their disease compared to 11% of women who completed their recommended treatment (36). Among women on AI therapy for risk reduction in early-stage breast cancer, one study estimates that nearly a quarter of patients discontinue therapy at a median time of 6 months due to AIMSS (37). While many patients are able to tolerate a switch to a different AI, the majority find insufficient relief and forgo continued AI therapy. Among patients who switched to a different AI due to adverse effects in this study, only 38% of patients were able to tolerate a second AI for a median of 13 months. Within the gynecologic oncology literature, one retrospective study found similar rates of AIMSS symptoms in women on AI therapy at equivalent doses for primarily advanced stage ovarian or uterine cancer, though only 5.0% of patients discontinued AI therapy due adverse effects (38). The authors of this study hypothesized that the gynecologic oncology sample was more likely to continue AI therapy since their sample population had more advanced disease on average than in breast cancer adjuvant therapy studies, however data is comparatively limited in the gynecologic oncology setting.

The prevalence of musculoskeletal symptoms has distinguished AIs from other endocrine therapies used in treatment of HR-positive breast cancer. Tamoxifen and AIs share numerous side effects including menopausal vasomotor and vulvovaginal symptoms. Compared to AIs, tamoxifen is associated with increased risk for venous thromboembolism, cerebrovascular accident, and endometrial cancer, though is relatively spared from musculoskeletal pain and has protective effects on bone mineralization (39). The significance of AIMSS is highlighted by the fact that arthralgias and bone health are among the most common reasons women switch from an AI to tamoxifen (40). Gonadotropin-releasing hormone (GnRH) agonists, most commonly used for ovarian function suppression in premenopausal women, are known to cause bone mineral loss predisposing to osteoporosis but uncommonly provoke musculoskeletal pain (41, 42). Moreover, fulvestrant, a selective estrogen receptor down-regulator (SERD) approved for use in advanced and metastatic hormone receptor positive breast cancer (43, 44), has an incidence of joint disorders comparable to or less than AIs (45–47) and its effects on bone density are not well defined (48).

Studies exploring risk factors for AIMSS have yielded inconsistent findings. Among the largest studies to identify correlative risk factors were retrospective analyses of the Arimidex Tamoxifen Alone or in Combination (ATAC) trial and the Intergroup Exemestane Study (IES) trial which discovered an association between AIMSS and body weight. ATAC and IES similarly found that BMI >30 and weight > 80 kg, respectively, correlated with increased risk for AIMSS (47, 49), with the association between obesity and AIMSS further supported by other study data (50). However, there may be a distinction in risk between overweight and obese. One cross-sectional survey found that women with BMI 25-30 had significantly fewer joint symptoms than both women with BMI >30 and BMI <25, indicating that being overweight but not obese may be protective (35). In contrast to these results, a number of smaller studies did not find BMI to be a significant risk factor (32, 51, 52). Some explanations for the associations with obesity have been proposed. Obesity and increased adipose tissue are known to be associated with increased aromatase activity and increased estrogen levels. A study of 44 women on AI therapy found that BMI correlated with higher baseline estradiol and estrone sulfate was associated with incomplete though still effective suppression of estrogen with AI therapy (52). Baseline estradiol and estrone sulfate levels in patients with BMI>35 were nearly three times that of women with BMI <25 and obese patients had more significant absolute decreases in estrogen levels compared to patients in lower BMI categories. The gravity of decrease in estrogen has been proposed as a possible mechanism for higher AIMSS risk in obese patients (47). Obesity is also a notable independent risk factor for osteoarthritis and carpal tunnel syndrome and thus may contribute to musculoskeletal symptoms through mechanisms irrespective of AI therapy (53–55) The exact relationship of AIMSS with obesity remains poorly understood.

Some data indicate that perimenopausal women are at higher risk for AIMSS than women who have been postmenopausal for a longer duration. A prospective and cross-sectional study found that women whose last menstrual period was within 5 years had higher rates of arthralgias while on AIs (32, 52). One proposed explanation argues that women who have more recently reached menopause have higher residual circulating estrogen and that AI therapy results in a more precipitous decline in estrogen from their baseline (52). Moreover, both the ATAC and IES trials found that prior hormone replacement therapy was a significant risk factor for musculoskeletal symptoms (35, 47, 49). This supports the notion that AIMSS onset may be related to the absolute drop and rate of change in estrogen levels from baseline.

Prior use of tamoxifen has not been clearly identified as a risk factor for AIMSS. Studies have been discordant with varying data suggesting prior tamoxifen use decreases risk (34, 56), increases risk (57), or is not associated with AIMSS (32, 58).

Results correlating AIMSS with taxane chemotherapy have similarly been mixed. Patients who received taxanes as part of their chemotherapy regimen were estimated to have four times the risk of developing AI-associated joint pain and stiffness in a cross-sectional survey (30, 34), and the implication of taxanes was supported by the ATAC trial, which reported higher rates of AIMSS in women who received chemotherapy (48). The association between AIMSS and taxanes however has not been well replicated in other studies (32, 49–51, 57–61). Taxane administration is recognized as an independent risk factor for arthralgias and myalgias further making the relationship with AIMSS unclear (62, 63). It remains unknown if taxanes and AIs have a synergistic role with regard to the development of arthralgia pain.

A recent systematic review and meta-analysis has looked at the protective role CDK4/6 inhibitors may provide against the development of AIMSS. CDK4/6 inhibitors are a novel class of drugs utilized in the treatment of metastatic HR+ breast cancer. The meta-analysis of 13 phase III trials involving patients on combined AI and CDK4/6 *versus* AI monotherapy demonstrated arthralgias in 1-47% of patients receiving AI monotherapy compared to a rate of 5.8-33.3% in those on combined treatment (64). Similarly lower rates were observed when comparing the AI monotherapy group to combination AI and CDK4/6 inhibitors for myalgias (2-23.7% *vs* 4.8-11.9%), back pain (7-32.9% *vs* 2.9-8.5%), and bone pain (7-32.9% *vs* 2.9-8.5%). Although promising, larger trials will be needed to clarify the role of CDK4/6 inhibitors in the mitigation of AIMSS.

Interestingly, it has been proposed that AIMSS may be a marker of improved outcomes with respect to decreased recurrence rates and improved disease-free and overall survival (65–68). While this association has not been consistently observed in all trials (49, 69), there has been speculation regarding this observation. It has been suggested that patients reporting adverse effects are more adherent to therapy (70) or alternatively that patients with AIMSS are benefiting from greater reductions in estrogen levels with enhanced efficacy against HR+ breast cancer (71).

## Manifestations of AIMSS

The musculoskeletal adverse events associated with AIMSS can be primarily classified into two major groups of 1) AI-induced bone loss and 2) AI-induced arthralgias (AIA). The clinical features and pathogenesis of these categories will be elaborated on in the following sections.

### AI-Induced Bone Loss

Several studies have established that treatment with AIs results in loss of bone density and increased fracture risk (71–73). A meta-analysis of seven randomized controlled trials (RCTs) involving 30,023 patients demonstrated that longer duration of AI use was associated with increased odds of developing bone fractures (OR = 1.47, 95% CI = 1.34 to 1.61, P <.001) (74). One potential mechanism is related to the hypoestrogenic state induced by AIs. In postmenopausal women, aromatase regulates estrogen which plays a role in the modulation of bone mass. As previously stated, AIs decrease estrogen concentrations by blocking aromatase (75). In post‐menopausal women, anastrozole, letrozole and exemestane lower the serum levels of estrogen by 81–94%, 88–98% and 52–72%, respectively (76).

**Figure 1:**
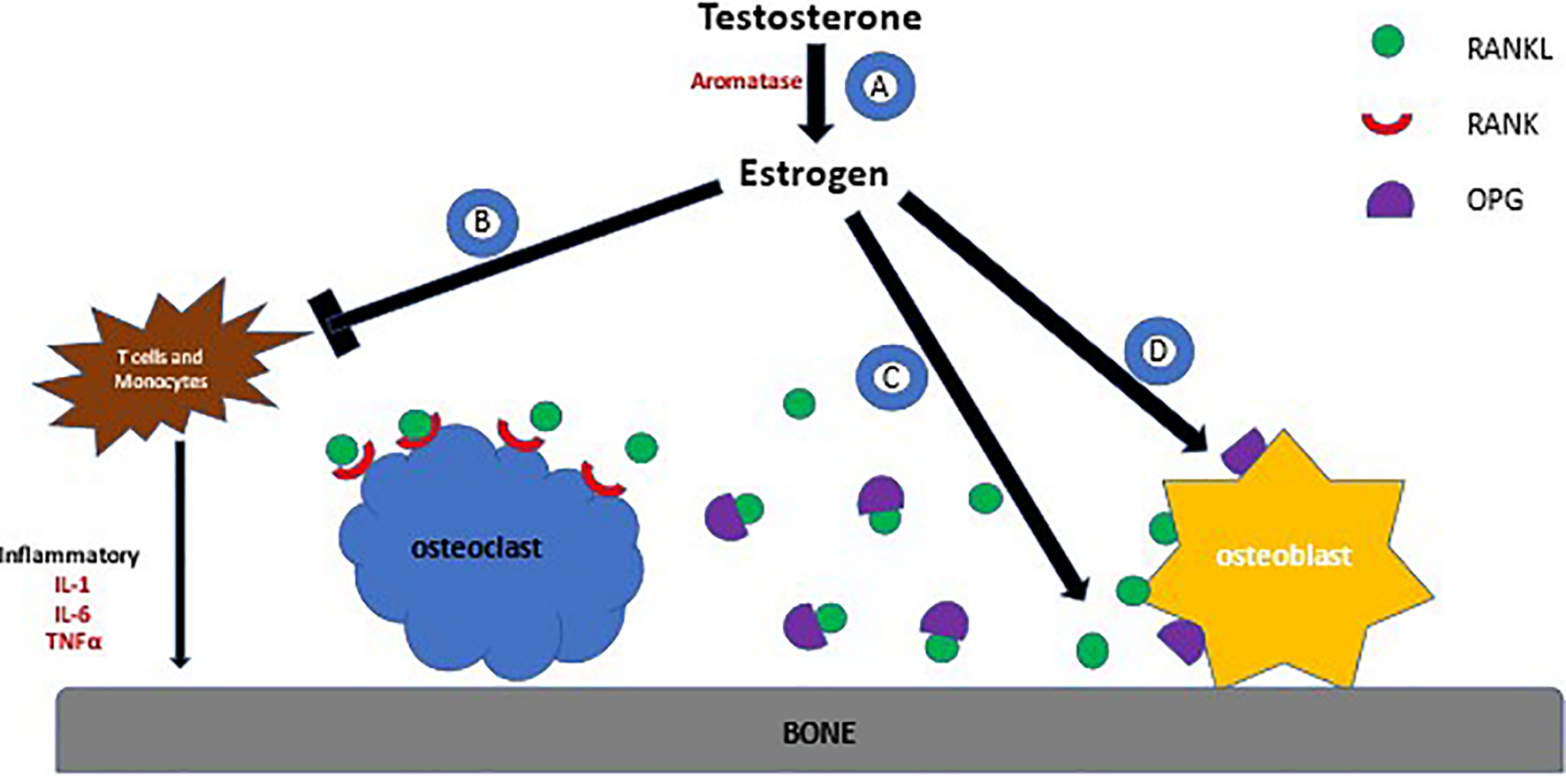
Effects of Estrogen on Bone Metabolism. **(A)** Testosterone is metabolized to make estrogen by aromatase. **(B)** Estrogen prevents the production of cytokines by T cells and monocyctes that are responsible for bone resporption **(C)** Estrogen decreases production of RANKL by osteoblasts, a protein that binds to the RANK receptor on the surface of osteoclasts. RANK stimulates the osteoclast to adhere to bone, activating bone resorption **(D)** OPG is produced by osteoblasts and acts as a decoy receptor for RANKL, thus preventing osteoclastsic activity.

At the cellular level, bone metabolism is a balance between osteoblastic and osteoclastic activity (**Figure 1**). Estrogen decreases the osteoblastic production of resorptive cytokines such as receptor activator of nuclear factor kappa B ligand (RANKL), colony‐stimulating factor‐1 (CSF-1), Interleukin-1 (IL-1), and tumor necrosis factor (TNF) resulting in an increase in osteoblast activity (77). Simultaneously, estrogen increases the production of osteoprotegerin (OPG) that is vital in inhibiting osteoclastogenesis and bone resorption. RANKL attaches to its receptor on the osteoclast surface and promotes cell differentiation, whereas OPG prevents this by binding to RANKL directly. Thus, in the absence of estrogen these mechanisms are hindered. Furthermore, estrogen deficiency is concomitantly associated with higher production of TNFα and RANKL by T cells and monocytes, resulting in increased bone resorption (78).

Recent evidence suggests that single-nucleotide polymorphisms (SNPs) in certain genes lead to AI-induced bone loss. In postmenopausal ER-positive breast cancer, SNPs in the genes encoding the estrogen receptors (ESR1 and ESR2), the expression of aromatase (CYP19A1), and CYP11A1 were predictors of decreased bone density (79). Additionally, a case-cohort genome-wide association study (GWAS) using samples from 1071 patients identified three SNPs in or near six genes (CTSZ, SLMO2, ATP5E, TRAM2, TRAM14A, MAP4K4) that were associated with increased risk of bone fractures in those receiving AIs (80). These genes displayed estrogen-dependent induction and their knockdown increased the expression of genes implicated in the development of osteoporosis.

### Aromatase Inhibitor-Induced Arthralgias

AIA is a common manifestation occurring in patients undergoing treatment with AI. Although there is no widely accepted definition for AIA a review proposed one that requires patients to meet all major criteria and at least three minor criteria to be met (**Table 1**) (81).

**Table 1:** Definition of AIA.

Major criteria	
- Currently taking AI therapy - Joint pain which has developed or worsened since starting AI therapy - Joint pain improves or resolves within 2 weeks of stopping AI therapy - Joint pain returns upon resuming AI
**Minor criteria**
- Symmetrical joint pains - Pain in hands and/or wrists - Carpal tunnel syndrome - Decreased grip strength - Morning stiffness - Improvement in joint discomfort with use or exercise

The AIA syndrome consists of symmetrical joint pain and stiffness most commonly affecting hands, wrists, knees, ankles, and shoulders, but can also involve central joints of the axial spine and pelvis. Other symptoms include decreased grip strength, myalgias, and extra-articular presentations such as carpal tunnel syndrome and trigger finger (82). A meta-analysis of twenty-one studies involving 13,000 patients showed the prevalence of AIA ranging from 20-74% (30). Although AIA can occur at any time after initiating AI, the median time to onset is approximately 6 weeks with peak symptoms at 6 months (59). Intolerance to AI therapy causes 20-30% of patients to discontinue treatment early, with 75% of them citing arthralgias as the primary reason (34). The arthralgias are typically relieved with discontinuation of AI therapy and recur with resumption of the AI.

Although not well understood, several mechanisms have been proposed for the development of AIA. Similar to AI-induced bone loss, estrogen deprivation has been proposed as a cause for the development of arthralgias. Aromatase is expressed in synovial cells and chondrocytes in articular cartilage (83). The estrogen produced here impacts chondrocyte formation through interactions with cytokines, adhesion molecules, and cell growth factors. Declining levels of estrogen result in increased production of proinflammatory cytokines such as interleukin-6 (IL-6) and IL-1 in articular chondrocytes resulting in joint pain and swelling (84). The chondroprotective role of estrogen was demonstrated in ovariectomized rats that demonstrated resorption of subchondral bone and accelerated degeneration of articular cartilage four weeks after surgery (85). With hormone replacement therapy, cartilage erosions were significantly reduced. Similarly, studies have demonstrated that hormone replacement with conjugated equine estrogens result in decreased joint pain, pain severity, and joint swelling in postmenopausal women (86).

SNPs in several genes have been linked to the development of AIA and myalgias. In one study of 254 BC patients on AI, increased musculoskeletal pain was associated with the SNP rs2073618 in OPG (87). In the B-ABLE cohort study, 343 women with AIA were found to have SNPs in the CYP17A1 (gene encoding for enzymes involved in estrogen metabolism) and VDR and CYP27B1 (genes encoding for enzymes involved in vitamin D signaling) (88). In another randomized clinical trial an inherited genetic variant involving an SNP in ESR1 (rs9322336) was associated with increased risk of musculoskeletal toxicity-related exemestane discontinuation (HR 5.0 (95% CI 2.1–11.8), p<0.0002) (89). Additionally, a study with 1049 women on AI were found to have an SNP (rs11648233) within the *HSD17B2* gene in the estrogen pathway which significantly increased odds of developing AIA (OR 2.21, 95% CI 1.55–3.16) (90).

Other possible mechanisms responsible for AIA relate to the effects of estrogen on the pain pathway. Aromatase and estrogen receptors are expressed in the brain and the spinal cord kappa-opioid analgesic system (91). Estrogen have anti-nociceptive properties therefore deprivation can decrease pain thresholds, which leads to increased pain perception in women treated with AIs. In one animal model, ovariectomized rats who were treated with letrozole demonstrated significantly reduced pain thresholds compared to those not treated (92). Furthermore, examination of the dorsal root ganglia of rats treated with letrozole long-term showed increased excitability of the small and medium-diameter sensory neurons, providing evidence that AIs can augment the pain response.

Joint stiffness and tenosynovitis of the hands or feet in the absence of systemic inflammation or autoimmune disease is a common presentation of AIMSS (93). Studies have demonstrated characteristic radiologic changes demonstrating the inflammatory changes associated with AIA (94, 95). In a study involving 12 women reporting severe AIA, ultrasound and MRI showed increased fluid in the tendon sheath surrounding the digital flexor tendons, thickening of the tendon sheath, and increased intra-articular fluid (96). Similar findings were reported in a larger study where musculoskeletal sonography demonstrated increased tendon thickness (p<0.001) and higher rates of joint effusions (p<0.033) in patients receiving AIs (97).

Another proposed mechanism is that AI therapy can lead to the induction of the autoimmune system. From the rheumatology literature, estrogen appears to play a key role in the pathogenesis of autoimmune disease. In preclinical studies, animal models of human rheumatoid arthritis induced an increase in proinflammatory cytokines IFN-γ and IL-12 and decreased levels of anti-inflammatory cytokines IL-4 and IL-10 secretion when treated with anastrozole (98). AI treatment has additionally been shown to suppress differentiation of naïve T cells to Tregs which is important in preventing the autoimmune response and stimulation of CD4+ T cells involved in the inflammatory response. Thus, AI treatment results in inflammatory changes by the cytokine-induced activation of fibroblasts, macrophages, and monocytes in the joint microenvironment and the CD4+ T cell infiltration into the synovial membrane (99). Similarly, clinical studies in breast cancer patients treated with 6 months of letrozole found a significant Treg cell down-regulation (100). Further implication of a AI induced autoimmune response is that discontinuation of AI treatment results in decreased arthralgias greater than two-third of patients and decrease in autoimmune markers including ANA and RF (101).

Other preclinical studies have looked at the role of aromatase in the development of autoimmune disease. In one study, aromatase-knockout (ARKO) mice developed inflammatory changes in salivary and lacrimal glands similar to human Sjogren’s syndrome which was further exacerbated by the administration of exemestane (102). The glands of the ARKO mice had a higher infiltration of white adipose tissue which expressed increased levels of proinflammatory cytokines and macrophages compared to the wild-type mice. These results suggest that aromatase may have a role in the pathogenesis of autoimmune disease such as Sjogren syndrome. Another potential biological pathway involves SNPs that created an estrogen response element near the 3’ end of the T-cell leukemia 1A (TCL1A) gene and was associated with increased musculoskeletal pain in women on adjuvant AI for breast cancer (103). Estradiol increases expression of TCL1A. The SNP increased expression of TCL1A in an estrogen-dependent manner which resulted in upregulation of IL-17RA expression and downregulated the expression of IL-17, IL-12, IL-12RB2 and IL-1R2. IL-17 is a key driver in the T-helper type 1 and 17 immune pathway in patients with autoimmune disease (104). Therefore the E2-dependent regulation of cytokine and cytokine receptor expression mediated by TCL1A might help explain the association of TCL1A and AIMSS. However, a small clinical study of 198 postmenopausal HR+ breast cancer patients was not able to find an association between patients with AIMSS and TCL1A polymorphisms (105).

### Aromatase Inhibitors: Similarities and Differences

In contrast to the first two generations of AIs, the third generation AIs demonstrate high potency by inhibiting ≥98% aromatase activity *in vivo*(106) and have been consistently found to suppress plasma estrogen levels >90% (107). Letrozole has been recognized to be more potent than anastrozole and exemestane at prescribed doses, though the clinical significance of this difference remains unclear (108–114). A randomized cross-over study of 12 patients found that letrozole 2.5 mg daily suppressed plasma levels of estrone, estradiol, and estrone sulfate greater than anastrozole 1 mg daily (114), and a subsequent study by the same researchers demonstrated that letrozole also suppressed estrone, estradiol, and estrone sulfate levels greater than anastrozole in breast cancer tissue in addition to plasma levels (115). Evidence of letrozole’s greater potency was further supported by a randomized cross-over study of 54 patients demonstrating significantly lower estradiol and estrone sulfate plasma levels in patients receiving letrozole *versus* anastrozole (116). In contrast to letrozole and anastrozole, there are limited data directly comparing the potency of exemestane’s estrogen suppressive effects with that of anastrozole and letrozole. Early studies showed that exemestane suppressed whole body aromatase activity and plasma levels of estrone, estradiol, and estrone sulfate *in vivo* to a similar degree as anastrozole and letrozole, though it was not compared directly with anastrozole or letrozole (115). A recent cross-over trial using exemestane and letrozole in the neoadjuvant setting discovered higher estrogen activity in serum samples during exemestane therapy than during letrozole therapy in 21 out of 26 patients, suggesting that letrozole suppresses estrogen greater than exemestane (117).

Letrozole’s modest but greater potency for estrogen deprivation has not been evidently associated with more severe AIMSS. A single-blind, crossover trial of 72 women whose HR+ breast cancer progressed on tamoxifen were randomized to either letrozole or anastrozole for four weeks and then crossed over to the other AI for another 4 weeks found significantly less reported joint pain in the letrozole arm than the anastrozole arm (3% *vs* 11%; p=0.025) and a greater preference for continued treatment with letrozole among participants (p<0.01) (118). Furthermore, the Articular Tolerance of Letrozole (ATOLL) study was a non-randomized prospective study that aimed to assess the effect of switching to a difference AI therapy after intolerable musculoskeletal symptoms. The study followed 179 patients who discontinued anastrozole due to AIMSS and were switched to letrozole after a 1-month anastrozole washout period. After 6 months, 71.5% of patients remained on letrozole while 28.5% of patients discontinued it due to persistent AIMSS. While AIMSS remained highly prevalent with reported rates of arthralgia (73.9%), myalgia (21%), arthritis (15.9%), tendinitis (14%), and polyalgic syndrome (12.7%), fewer musculoskeletal symptoms were reported than when on anastrozole and patients had significantly improved pain and QoL survey results after switching to letrozole. A shorter period of time until discontinuation of anastrozole was predictive of letrozole discontinuation (p=0.04). The ATOLL study showed that patients may be able to tolerate AI therapy longer if switched from anastrozole to letrozole though the study was not designed to clearly answer if letrozole or anastrozole are more likely to cause AIMSS.

A series of studies have demonstrated no difference in AIMSS between anastrozole and letrozole. One randomized multicenter trial of 713 women with advanced breast cancer found no significant differences in tolerability or safety between anastrozole and letrozole (119). The Anastrozole *Versus* Letrozole: Investigation into Quality of Life and Tolerability (ALIQUOT) study was an open-label crossover trial involving 181 HR+ breast cancer patients randomized to 12 weeks of letrozole followed by 12 weeks of anastrozole or vice versa designed to detect differences in adverse effects (116). In the trial, AIMSS were significantly associated with the second 12-week period in both arms indicating that the time from starting an AI rather than the difference between letrozole or anastrozole best explained joint pain development. Letrozole and anastrozole had comparable tolerability with similar rates of joint pain and quality of life measurements. Moreover, the Femara *Versus* Anastrozole Clinical Evaluation (FACE) trial, a randomized, open-label trial comparing adjuvant letrozole to anastrozole in node-positive HR+ breast cancer for 5 years or until disease recurrence, demonstrated similar rates of AIMSS with grade 3-4 adverse events of arthralgia 3.9% and 3.3% and myalgia 0.8% and 0.7% in letrozole and anastrozole arms, respectively (120). Additionally, one retrospective review of 141 patients in Japan comparing letrozole to anastrozole found no difference in frequency or time to onset of joint symptoms, though the anastrozole group had a shorter time to onset of painless joint symptoms (p=0.022), such as joint stiffness or decreased joint motion (121).

Data exploring comparative differences in joint pain between exemestane use with other AIs is limited and did not reveal significant differences (122, 123). While there is a paucity of literature comparing joint symptoms between exemestane against anastrozole and letrozole, studies exploring the comparative effects of exemestane on bone turnover and bone mineral density are more robust. Exemestane has been hypothesized to have less adverse effects on bone density than the non-steroidal AIs anastrozole and letrozole. Exemestane’s steroidal structure affords it distinct endocrine properties. The exemestane metabolite 17-hydroexemestane exhibits androgenic activity suggested to stimulate bone formation. One animal model showed that exemestane decreased serum markers of bone turnover pyridinoline and osteocalcin and resulted in greater bending strength and trabecular bone volume in ovariectomized rats (124). In human biomarker studies, serum procollagen type 1 N-terminal propeptide and urine N-telopeptide, markers of bone formation, were significantly increased in patients on exemestane compared to a non-steroidal AI (125, 126). However, some studies have not demonstrated significant differences in markers of bone turnover between exemestane, anastrozole, and letrozole arms (127). There is evidence that exemestane use results in less BMD loss compared to letrozole and anastrozole at 24 months of therapy (128, 129), though it remains to be seen if fewer patients on exemestane develop fractures. The MA.27 trial comparing exemestane with anastrozole in the adjuvant setting identified less reported osteoporosis in patients randomized to exemestane, 31% *vs* 35% in the exemestane and the anastrozole arm (p=0.001), respectively (130), however further analysis within the MA.27 cohort showed no significant differences in BMD between exemestane and anastrozole at 24 months (130). While exemestane results in BMD loss in postmenopausal women with breast cancer (131), it may cause slower BMD loss than non-steroidal AIs.

To our knowledge, there is no head-to-head study comparing anastrozole, letrozole, and exemestane designed to explore differences in the manifestations and severity of AIMSS in patients on AI therapy. Such a trial would help clarify differences in AI therapy by helping to answer if letrozole’s more potent estrogen suppression leads to greater severity of AIMSS symptoms and if exemestane’s steroidal structure results in fewer fractures. With greater understanding of the mechanisms of AIMSS, HR+ breast cancer therapies with fewer musculoskeletal symptoms can be developed. Advancements in pharmacologic research increase optimism for the design of more tolerable therapies targeting aromatase that spare musculoskeletal tissues from toxicities of estrogen deprivation (128, 132).

## Management of AIMSS

### Pharmacological Therapy

Nonsteroidal anti-inflammatory drugs and acetaminophen can be used for pain control in the short-term. Opioids should not be used in the management of pain. There is no single mitigation strategy that has been found to be effective for AIMSS. Several pharmacological and complementary modalities have been studied with mixed results (**Table 2**).

**Table 2:** Studies Involving Pharmacological Management of AIMSS.

Intervention Author	Study Type	Number of patients	Study Arms	Outcomes
**Testosterone**				
Birrell et al. (134)	RCT	90	Arm 1: Placebo	VAS scores decrease 70% in testosterone arm *versus* 35% in placebo arm
			Arm 2: Testosterone 80 mg	
Cathcart-Rake et al. (135)	RCT	208	Arm 1: Placebo Arm 2: Testosterone 120 mg	No difference between BPI-AIA pain scores between arms
**Duloxetine**				
Henry et al. (136–138)	RCT	289	Arm 1: Duloxetine 30mg daily x 1 week then 60mg daily x11 weeks then 30mg daily x1 week	In obese patients, reduction in BPI-SF pain scores compared to placebo.
				In non-obese patients, no reduction in BPI-SF pain scores compared to placebo.
			Arm 2: Placebo	
**Switch AI**				
Briot et al. (139)	Prospective	179	Arm 1: Patients who developed AIMSS on anastrozole switched to letrozole	At the end of 6 months, 71.5% of patients adhered to second AI
Ribi et al. (140)	RCT	4884	Arm 1: Continuous letrozole (2.5 mg daily for 5 years)	Improved quality of life (QoL) scores including in musculoskeletal pain in group receiving intermittent letrozole *versus* continuous letrozole (mean score change=3; 95% CI: 0-6; p=0.023
			Arm 2: Intermittent use (2.5 mg daily for 9 months followed by a 3-month break in years 1-4 and then 2.5 mg daily in year 5	
Kadakia et al. (165)	Prospective	83	Arm 1: Patients who developed AIMSS on letrozole switched to exemestane	At the end of 6 months, 62% of patients adhered to second AI
			Arm 2: Patients who developed AIMSS on exemestane switched to letrozole	
**Bisphosphonates**				
Santa-Maria et al. (145)	Prospective	59	Arm 1: Zoledronic acid 4mg IV prior to AI then at 6 months	At the end of 1 year, 37% of patients reported AIA symptoms (defined by an increase in VAS and/or HAQ-II) compared to 67% of matched controls from ELPh trial
**Diuretics**				
Alhanafy et al. (147)	Prospective	50	Arm 1: Oral Furosemide 20mg + oral spironolactone 50 mg daily	At the end of 4 weeks, 7% of patients on diuretics have arthralgia compared to 16% on placebo (p=0.01)
**Steroids**				
Kubo et al. (148)	Prospective	27	Arm 1: Oral prednisolone daily x 1 week	At 1 week, 67% patients report improved symptoms using VAS. At 2 months, 52% patients continue to report improved symptoms.
**Vitamin D**				
Prieto-Alhambra et al. (153)	Prospective	260	Arm 1: Oral Vit D3 16000 IU every 2 weeks + oral calcium 1 g daily + oral Vit D3 800 IU daily	After 1 year of AI treatment, patients able to achieve 25-OHD levels ≥ 40 ng/mL had reduced AI-associated bone loss compared to patients with 25-OHD levels < 30 ng/mL (p = 0.005)
Khan et al. (154)	Prospective	60	Arm 1: Oral Vit D3 50000 IU weekly in patients with 25-OHD levels ≤ 40ng/mL	After 16 weeks of treatment with letrozole, patients who achieved 25-OHD levels > 66 ng/mL reported no disability compared to patients who achieved 25-OHD levels ≤ 66 ng/mL (52% *vs* 19%; p=0.026)
			Arm 2: Oral Vit D3 600 IU daily + calcium 1200mg daily in patients with 25-OHD levels > 40ng/mL	
Shapiro et al. (155)	RCT	113	Arm 1: Oral Vit D3 600 IU x 6 months	No significant difference between both arms based on BCPT-MS scores (p=0.38)
			Arm 2: Oral Vit D3 4000 IU x 6 months	
Rastelli et al. (166)	RCT	60	Arm 1: Oral Vit D2 50000 IU x 8-16 weeks then monthly for 4 months	At 2 month follow up, pain scores measured by FIQ and BPI-SF lower in patients receiving Vit D2 (p=0.02). At 6 month follow up, no statistical difference noted between both arms.
			Arm 2: Placebo	
Niravath et al. (167)	RCT	93	Arm 1: Oral Vit D3 50000 IU weekly x 12 weeks then 2000 IU daily x 40 weeks	Study terminated due to no statistical difference between two arms at 12 weeks (54% *vs*. 57%)
			Arm 2: Oral Vit D3 800 IU daily x 52 weeks	
**Glucosamine Chondroitin**				
Greenlee et al. (168)	Prospective	53	Arm 1: Oral Glucosamine 1500mg + oral Chondroitin 1200mg daily x 24 weeks	At the end of 24 weeks, improvement in pain scores in 46.2% of patients as measured by BPI, WOMAC, and M-SACRAH
**Omega 3 Fatty Acids**				
Hershman et al. (157)	RCT	249	Arm 1: Oral O3-FA 3.3 g daily	At 12 weeks and 24 weeks, no statistical difference in pain scores based on BPI, WOMAC, and M-SACRAH
			Arm 2: Placebo	
Lustberg et al. (169)	RCT	44	Arm 1: Oral O3-FA 4.3 g daily	At 12 weeks and 24 weeks, no statistical difference in pain scores based on BPI. Quality of life score based of FACT-ES significantly decreased in placebo compared to treatment group (p=0.06) at 12 weeks but not at 24 weeks
			Arm 2: Placebo	
Shen et al. (170)	Analysis of subpopulation of obese patients from Hershman et al.	110	Arm 1: Oral O3-FA 3.3 g daily	In obese patients, pain scores lower in arm 1 compared to arm 2 based on BPI, WOMAC, and M-SACRAH
			Arm 2: Placebo	

AI, aromatase inhibitor; AIA, aromatase inhibitor associated arthralgia; BCPT-MS, Breast Cancer Prevention Trial-Musculoskeletal Symptoms Subscale; BPI-AIA, brief pain inventory; ELPh trial, Exemestane and Letrozole Pharmacogenomics; FACT-ES, Functional Assessment of Cancer Treatment-Endocrine Symptoms; HAQ-II, Health Assessment Questionnaire; M-SACRAH, Modified Score for the Assessment and quantification of Chronic Rheumatoid Affections of the Hands; VAS, visual analogue score; WOMAC, Western Ontario and McMaster Osteoarthritis Index.

### Testosterone

Testosterone and dihydrotestosterone, have an anti-inflammatory effect on joints minimizing joint pain and damage (133). In a phase II trial, ninety women on adjuvant anastrozole were randomized to receive placebo, 40mg of testosterone undecanoate, or 80mg of testosterone undecanoate (134). Patients receiving testosterone reported pain reduction at 3 months with 43% in the testosterone 40mg group (p=0.06) and 70% in the testosterone 80mg group (p=0.04). Additionally, testosterone levels stabilized within a physiologic range at 3 months and did not result in significantly increased estradiol concentrations, an important finding considering that HR+ breast cancers also express androgen receptors. In the subsequent A221102 trial, 208 postmenopausal women experiencing moderate-to-severe arthralgias while taking adjuvant AI were randomized to receive testosterone or placebo (135). Two thirds of patients reported improvement in joint pain at 3 months although there was no significant difference between the two groups. The discrepancy between the two studies may partially be explained by the fact that the second study utilized topical testosterone gel that may not achieve the same systemic concentrations as the oral formulation.

### Duloxetine

Duloxetine is a serotonin-norepinephrine reuptake inhibitor used to treat depression and chronic pain conditions. In a randomized control trial, 300 patients with AIMSS received either duloxetine (30 mg daily for 1 week, 60 mg for week 2-11, and then 30 mg daily for week 12) or placebo (136). At week 6 of treatment, patients receiving duloxetine had a clinically significant decrease in joint pain score (> 2 points) compared to placebo (68% *versus* 49%; p=0.003). By 12 weeks, the average joint score was 0.82 points lower in the duloxetine-treated group compared to placebo. However, once the drug was discontinued, the pain scores were equivalent in the two arms, suggesting that duloxetine exerts an analgesic effect rather than a reversal in the disease process. In addition, rates of adverse events were higher in the group receiving duloxetine (78% *versus* 50%). Although duloxetine decreases AIMSS, benefit is only derived for the treatment duration and the adverse effects may make it intolerable for some patients. However, in the SWOGS1202 randomized control trial, 289 were randomized to receive duloxetine *versus* placebo (137). Patients receiving duloxetine were more likely to report more beneficial than placebo (73.3% *vs* 41.8%, respectively; 95% CI for difference = 15.4-47.2 percentage points). Further sub-analysis revealed that obese patients (BMI ≥ 30 kg/m^2^) obtained more analgesic benefits than the non-obese with a significant reduction in pain scores obese (-2.73 *vs* -1.64 points; P = .003). Conversely, in the nonobese patients, the reduction in the mean average pain score was similar in the 2 cohorts (-2.46 *vs* -2.34 points; P = .75) (138).

### Switching AIs or Intermittent Dosing of AIs

Another clinical approach suggests benefit from alternating between AI agents within the same class. In the prospective, non-randomized ATOLL trial, 179 patients who had discontinued anastrozole due to musculoskeletal symptoms received letrozole (139). At the end of the 6-month period, 72% of patients continued the letrozole with the remaining discontinuing treatment secondary to severe joint pain. Furthermore, among patients who continued letrozole, 74% continued to experience arthralgias, myalgias, arthritis, or tendinitis. The phase III SOLE trial investigated the intermittent use *versus* continuous use of letrozole. Postmenopausal women who had completed 4-6 years of adjuvant endocrine therapy and were clinically disease free of breast cancer were randomized to receive either continuous letrozole (2.5 mg daily for 5 years) or intermittent use (2.5 mg daily for 9 months followed by a 3-month break in years 1-4 and then 2.5 mg daily in year 5) (140). Although the primary outcome of disease-free survival (DFS) did not improve between the groups, patients receiving intermittent letrozole reported better quality of life (QoL) scores including in musculoskeletal pain (mean score change=3; 95% CI: 0-6; p=0.023) (141). The rates of adherence of letrozole were similar between both groups which could be the result of enrolling patients who had previously tolerated 4-6 years of endocrine therapy. Therefore, they may not have experienced significant AIMSS leading to discontinuation of the drug. These results suggest that intermittent administration of AIs can result in increased tolerability of the drug without significantly affecting disease outcomes.

### Bisphosphonates and Denosumab

Bisphosphonates have an integral role in the treatment of osteoporosis given their ability to decrease bone loss and increase bone density. Several interdisciplinary cancer and bone societies have developed an algorithm for the management of AI-associated bone loss (142). In all patients initiating AI treatment, lifestyle changes such as diet rich in calcium, weight-bearing exercise, limitation in alcohol and smoking cessation are recommended. All patients should be monitored for fracture risk and bone marrow density (BMD) every 1-2 years. Risk factors associated with increased fracture risk should be identified which include age > 65 years, T-score < 1.5, smoking, BMI<24, family history of hip fractures, personal history of fragility fracture above age 50, and oral glucocorticoid use >6 months. For patients with a T-score < -2.0 and/or more than two of the above risk factors is present, an anti-resorptive agent, such as bisphosphonates or denosumab, should be utilized. In an observational cohort study involving 36,472 breast cancer patients, fracture risk in AI users was > 40% compared to tamoxifen (143). However, amongst patients on AIs at high risk of fracture, bisphosphonate-treated patients had an HR 0.73 [95% CI, 0.51 to 1.04] and SHR 0.69 [95% CI, 0.48 to 0.98] for fractures compared to those not on concomitant bisphosphonates. In the ABCSG-18 trial, AI-treated patients who received denosumab for 3 years showed a lower incidence of fractures and a significant increase of the femoral neck and lumbar spine BMD compared to patients receiving placebo (144).

Based on the efficacy of bisphosphonates in decreasing bone loss, studies have investigated their role in the management of AIA. In the single-arm Zoledronic Acid Prophylaxis (ZAP) trial, 59 postmenopausal breast cancer patients received zoledronic acid concomitantly with letrozole for six months (145). Compared to historical controls from the Exemestane and Letrozole Pharmacogenomics (ELPh) trial, there was a significant decrease in AIA within the zoledronic acid group (AIA incidence: 37% *versus* 67%; p<0.001).

### Diuretics

A retrospective analysis of women treated with adjuvant AI therapy showed that women who were on chronic diuretic treatment for heart disease or hypertension were less likely to have symptoms of arthralgia, muscular or skeletal stiffness (6.97% *versus* 15.85%, *P* = 0.01), suggesting that fluid retention within joints may play a role in AI-induced arthralgia (146). In a phase II trial, 50 women were randomized to receive diuretic or placebo for 4 weeks (147). After the treatment period, the modified Western Ontario and McMaster Universities osteoarthritis (WOMAC) index for lower limb improved significantly (6.0 v 10; P < 0.001), in addition to improvement in the mean WOMAC stiffness score (2.3 v 3.9; P = 0.002), the mean WOMAC functional score (8.7 v 15; P < 0.001), and the total WOMAC score (17 v 29; P < 0.001).

### Steroids

In a single-arm study, 27 patients with AIMSS were administered 5 mg of prednisolone daily for one week (148). Pain scores improved in 67% of patients at one week with one half of patients still reporting analgesic benefit at two months follow-up. Further studies need to be done to examine the long-term benefit of this intervention in these patients.

### Vitamin D Supplementation

Patients receiving AIs are frequently vitamin D deficient most likely due to the role estrogen has in the activation of vitamin D and its receptor (149). Vitamin D is essential for calcium absorption and bone mineralization, which is positively associated with bone mineral density. A number of studies have suggested the importance of vitamin D in improving muscle strength and function (150–152). Therefore, correcting a vitamin D deficient state may aid in alleviating musculoskeletal symptoms associated with AI-treatment. In a prospective cohort study involving 290 breast cancer patients received 800 IU of vitamin D_3_ daily (16000 IU of vitamin D_3_ every 2 weeks given in addition to those with baseline 25(OH)D concentration <30 ng/ml) (153). Although there was an increase in joint pain in the cohort based on visual analog scale (VAS) for joint pain (mean 1.16 points SD 2.66; P < 0.001), the increase was significantly less (p=0.02) in those patients who were able to reach 25(OH)D concentrations of ≥40 ng/ml. Similar findings were reported in a cohort study that demonstrated differences in pain scores between patients receiving vitamin D_3_ supplementation who achieved 25(OH)D concentrations of ≥ 66 ng/ml (19% *versus* 52%; p=0.026) (154). Conversely, in a recent trial that randomized breast cancer patients experiencing AIMSS to receive either 600IU D_3_ or 4000 IU D_3_, there was no statistical significance in the primary endpoint which was a change in AIMSS score from baseline (155). Another important issue is that certain breast cancer subtypes express VDR and long term clinical data will be needed to ensure that vitamin D supplementation does not have a negative therapeutic impact. Further research is needed to examine the utility of vitamin D supplementation as a way to ameliorate AIMSS.

### Omega-3 Fatty Acids

Omega-3 fatty acids (O3-FA) has demonstrated efficacy in decreasing joint pain, the number of swollen joints, and the use of NSAIDs in patients with inflammatory arthritis, prompting an investigation with regard to its role in AIMSS management (156). In the SWOG S0927 trial, 262 women were randomized to receive O3-FA *versus* placebo for 24 weeks. At the end of the study period, AIMSS symptoms as measured with the Brief Pain Inventory-Short Form (BPI-SF) decreased in the O3-FA group compared to placebo group (mean score=2.22 *versus* 1.81; p<0.001) (157). A *post-hoc* analysis demonstrated a significant improvement in pain scores in obese patients (BMI≥ 30kg/m^2^) receiving O3-FA *versus* placebo; a difference absent among non-obese patients (BMI < 30kg/m^2^). These findings may be attributed to the anti-inflammatory effect of O3-FAs on adipose tissue, which is thought to be a source of inflammatory mediators.

## Complementary Therapy

### Physical Activity

Exercise can have multiple benefits in the management of AIMSS. Similar to its role in osteoporosis, exercise can help increase bone density (158). Moreover, it can increase the circulation of body fluid to tissues and increase the volume of skeletal muscle making physical activity easier (159). Exercise may also increase the pain threshold by improving the range of motion and muscle strength of patients experiencing musculoskeletal symptoms (160). An effective exercise program refers for the prevention and improvement of AIMSS includes aerobic exercise, resistance exercise, or a combination of both. It can involve activities such as yoga, walking, and swimming. Exercise intensity should be guided by an exercise trainer at a safe and comfortable pace or no more than 80% of heart rate reserve. A meta-analysis of 9 studies involving 743 participants evaluated the effect of aerobic exercise in alleviating AIMSS. The duration of interventions ranged from 6 weeks to 12 months with at least 120 minutes/week of exercise prescribed. Aerobic exercise was performed in all studies, five of which included resistance exercise. Results demonstrated an improvement in scores of pain (p=0.006), stiffness (p=0.01), and grip strength (p=0.002) and an overall improvement in quality of life, regardless of the form of exercise program undertaken (161). Another form of exercise providing relief was yoga. In a study, 95 breast cancer patients on AI or tamoxifen were randomized to undergo standard care *versus* 4 weeks of yoga. At baseline, the AI group reported higher levels of myalgias and arthralgias; however, amongst those in the yoga group, there was improved scores at the end of the intervention period with 90% reporting reduced severity of symptoms (162).

### Acupuncture

Acupuncture is a non-pharmacologic modality used in the treatment of a variety of pain conditions. Small trials have investigated its role in the treatment of AIMSS. In a RCT, 226 patients were randomized to true acupuncture, sham acupuncture, or no treatment for 6 weeks (163). The BPI-WP at the end of the study period demonstrated a decrease by 2 points in the true acupuncture group compared to 1 point in the other two groups. The pain-reporting guidelines describe the clinical meaning of an individual’s response, with studies suggesting that a reduction of 2 points on an 11-point scale (or 30%) represents a clinically significant improvement (164). In this study, 58% of patients in the true acupuncture group reported at least a 2-point improvement compared to 33% in the sham group and 31% in the no treatment group. Currently, a RCT investigating a novel treatment of auricular point acupuncture as a low-cost intervention in the management of AIMSS (NCT03697200) is underway.

## Future Directions

Despite the central role of AIs in the chemoprevention and management of HR+ breast cancer in postmenopausal women, significant musculoskeletal side effects have led to premature discontinuation of therapy in patients. AIMSS encompasses bone loss, arthralgias, and autoimmune rheumatologic disease. Although there have been several mechanisms proposed for AIMSS, it remains challenging to predict which patients will develop musculoskeletal symptoms. In the B-ABLE study (NCT03811509), patients are randomized to receive bisphosphonates or denosumab concurrently with AI. The aim of this trial is to identify the deleterious effects of AIs on bone microarchitecture by dual energy x-ray absorptiometry (DEXA), lumbar spine Rx, Trabecular Bone Score (TBS) and bone mineral strength (BMSi) for early identification of patients at risk for developing AI-bone loss. Evaluation of cartilage degradation markers (C-telopeptide II, Procollagen type 2 N-terminal propeptide) may provide valuable information to identify both patients at risk, and further serve as objective markers for monitoring the development of AIMSS. Recently, there has been growing interest in potential genetic determinants leading to AIMSS. An active cohort study involving 1000 participants is underway to further elucidate the role of other SNPs in cytochrome P450 enzymes (CYP), glucuronosyltransferases (UGT), Vitamin D, serotonin and other receptors that may be associated with discontinuation of treatment due to the development of severe AIMSS (NCT01824836). The primary goal is to develop a gene signature that can help identify patients at risk for developing severe AIMSS. Additionally, there are a number of ongoing trials focusing on potential AIMSS management strategies including auriculotherapy (NCT03096041), physical therapy (NCT04560699), curcumin (NCT03865992), cannabidiol (NCT04754399), and tai chi (NCT04716920). Ultimately, a deeper understanding of the underlying mechanisms of AIMSS is needed to address and develop effective therapeutic strategies for the musculoskeletal adverse effects of AI therapy.

## Author Contributions

All authors contributed to the article and approved the submitted version.****

## Conflict of Interest

The authors declare that the research was conducted in the absence of any commercial or financial relationships that could be construed as a potential conflict of interest.

## Publisher’s Note

All claims expressed in this article are solely those of the authors and do not necessarily represent those of their affiliated organizations, or those of the publisher, the editors and the reviewers. Any product that may be evaluated in this article, or claim that may be made by its manufacturer, is not guaranteed or endorsed by the publisher.
